# *Stieleria varia* sp. nov., isolated from wood particles in the Baltic Sea, constitutes a novel species in the family *Pirellulaceae* within the phylum *Planctomycetes*

**DOI:** 10.1007/s10482-020-01456-9

**Published:** 2020-08-14

**Authors:** Frank Surup, Sandra Wiegand, Christian Boedeker, Anja Heuer, Stijn H. Peeters, Mareike Jogler, Mike S. M. Jetten, Manfred Rohde, Christian Jogler, Nicolai Kallscheuer

**Affiliations:** 1 Microbial Drugs Department, Helmholtz-Centre for Infection Research, Brunswick, Germany; 2 German Centre for Infection Research, Partner Site Hannover-Braunschweig, Brunswick, Germany; 3 Institute for Biological Interfaces 5, Karlsruhe Institute of Technology, Eggenstein-Leopoldshafen, Germany; 4 Leibniz Institute DSMZ, Brunswick, Germany; 5 Department of Microbiology, Radboud University, Nijmegen, The Netherlands; 6 Department of Microbial Interactions, Institute of Microbiology, Friedrich Schiller University, Jena, Germany; 7 Central Facility for Microscopy, Helmholtz Centre for Infection Research, Brunswick, Germany

**Keywords:** Marine bacteria, Biotic surfaces, Budding bacteria, Stieleriacines, Secondary metabolites

## Abstract

Species belonging to the bacterial phylum *Planctomycetes* are ubiquitous members of the microbial communities in aquatic environments and are frequently isolated from various biotic and abiotic surfaces in marine and limnic water bodies. Planctomycetes have large genomes of up to 12.4 Mb, follow complex lifestyles and display an uncommon cell biology; features which motivate the investigation of members of this phylum in greater detail. As a contribution to the current collection of axenic cultures of Planctomycetes, we here describe strain Pla52^T^ isolated from wood particles in the Baltic Sea. Phylogenetic analysis places the strain in the family *Pirellulaceae* and suggests two species of the recently described genus *Stieleria* as current closest neighbours. Strain Pla52n^T^ shows typical features of members of the class *Planctomycetia*, including division by polar budding and the presence of crateriform structures. Colonies of strain Pla52n^T^ have a light orange colour, which is an unusual pigmentation compared to the majority of members in the phylum, which show either a pink to red pigmentation or entirely lack pigmentation. Optimal growth of strain Pla52n^T^ at 33 °C and pH 7.5 indicates a mesophilic (i.e. with optimal growth between 20 and 45 °C) and neutrophilic growth profile. The strain is an aerobic heterotroph with motile daughter cells. Its genome has a size of 9.6 Mb and a G + C content of 56.0%. Polyphasic analyses justify delineation of the strain from described species within the genus *Stieleria*. Therefore, we conclude that strain Pla52n^T^ = LMG 29463^T^ = VKM B-3447^T^ should be classified as the type strain of a novel species, for which we propose the name *Stieleria varia* sp. nov.

## Introduction

*Planctomycetes* is a phylum of mostly aquatic bacteria, which can be found in various limnic and marine water bodies. Together with the medically and biotechnologically relevant phyla *Chlamydiae* and *Verrucomicrobia* and other sister phyla, the phylum *Planctomycetes* constitutes the PVC superphylum (Rivas-Marín and Devos 2018; van Niftrik and Devos 2017; Wagner and Horn 2006). Due to several presumptively eukaryotic characteristics (Fuerst and Sagulenko 2011), Planctomycetes were initially considered to be exceptions to the typical bacterial cell plan. However, with the introduction of novel microscopic techniques and the development of genetic tools for Planctomycetes (Jeske et al. 2016; Jogler et al. 2011; Rivas-Marín et al. 2016), several of their eukaryote-like characteristics have been reassessed. For example, the proposed intracellular compartments turned out to be rather invaginations of the cytoplasmic membrane (Boedeker et al. 2017). The identification of peptidoglycan in Planctomycetes (Jeske et al. 2015; Van Teeseling et al. 2015) led to the reinterpretation of their cell envelope architecture as being similar to that of Gram-negative bacteria (Devos 2014). Nevertheless, certain aspects of their cell biology are still exceptional. All characterised Planctomycetes lack ‘canonical’ divisome proteins including the otherwise universal FtsZ (Jogler et al. 2012; Rivas-Marin et al. 2020). Members of *Planctomycetia*, the class with the currently highest number of described species within the phylum *Planctomycetes*, divide by budding, whereas species belonging to the classes *Phycisphaerae* and *Candidatus* Brocadiae divide by binary fission (Wiegand et al. 2020).

The lifecycle of most Planctomycetes is complex and involves alternation between sessile cells attached to various abiotic and biotic aquatic surfaces, and flagellated swarmer cells (Faria et al. 2018; Lage et al. 2019). The sessile cells bud to form flagellated swarmer cells, which swim and relocate before settling down to attach and begin reproduction. In this context, Planctomycetes were found to be frequent colonisers of macroalgae (Bengtsson and Øvreås 2010; Faria et al. 2018; Lage and Bondoso 2014) and can even be the dominating phylum in microbial communities on biotic surfaces. For example, as recently shown, Planctomycetes can account for more than 80% of the bacterial community in seagrass meadows in the Mediterranean Sea (Kohn et al. 2020). Such findings appear counterintuitive when taking into account that the growth rates of Planctomycetes are often lower than those of many of their natural bacterial competitors occupying the same ecological niches (Frank et al. 2015). The observation that, despite slower growth, Planctomycetes can be abundant members in marine microbial communities led to the hypothesis that they apply different strategies to compensate for the disadvantage in growth speed, although most of these stategies remain undiscovered. It has been assumed that these strategies may involve the ability to produce bioactive secondary metabolites (Kallscheuer et al. 2019b; Panter et al. 2019), the observed resistance against several antibiotics (Cayrou et al. 2010; Godinho et al. 2019) and/or a metabolism well-adapted to digestion of phototroph-derived compounds, including complex polysaccharides (Wecker et al. 2009; Wegner et al. 2013).

Recently, a class of *N*-acylated tyrosine derivatives, designated stieleriacines, has been identified in the Planctomycete *Stieleria maiorica* Mal15^T^, which can influence the microbial community composition of biofilms inhabited by this species (Kallscheuer et al. 2020a). Structurally related compounds of the same class were also found in the closely related species *Stieleria neptunia* (Sandargo et al. 2020). In silico genome analyses indicate that the ability to produce secondary metabolites is widespread in the phylum, in particular in the class *Planctomycetia*. The hitherto investigated planctomycetal genomes feature sizes of up to 12.4 Mb (Ravin et al. 2018) and between 1 and 13 putative secondary metabolite-associated gene clusters were identified during in silico genome analyses (Wiegand et al. 2020). These clusters are similar to previously investigated clusters, e.g. those found in Actinobacteria, and may be similarly involved in the biosynthesis of non-ribosomal peptides, polyketides, terpenes, bacteriocins and others. Consequently, Planctomycetes are considered to be untapped producers of small molecules with potential therapeutically useful bioactivities (Calisto et al. 2019; Graça et al. 2016; Jeske et al. 2016).

To extend our knowledge on Plantomycetes in general and the genus *Stieleria* in particular, we herein characterise strain Pla52n^T^ by using physiological, microscopic, genomic, and phylogenetic methods. Based on these analyses, we propose that strain Pla52n^T^ constitutes the third species of the recently described genus *Stieleria*.

## Materials and methods

### Isolation of strain Pla52n^T^ and cultivation

Strain Pla52n^T^ was isolated from wood particles placed in an incubator and stored for two weeks (August–September 2014) at a depth of 2 m in the Baltic Sea, below a landing stage at Heiligendamm (‘Seebrücke Heiligendamm’, 54.146 N 11.843 E) (Oberbeckmann et al. 2018). In the laboratory, biofilms formed on the wood particles were removed by incubation with β-galactosidase (2 mg/mL, 30 °C, pH 4.7) for 30 min and subsequent sonication for 10 min at 30 °C. M1 medium buffered with 4-(2-hydroxyethyl)-1-piperazineethanesulfonic acid (HEPES) and supplemented with *N*-acetyl glucosamine (NAG) and artificial seawater (ASW) (designated M1H NAG ASW medium) (Boersma et al. 2019) was used for the cultivation. The medium was solidified with 8 g/L gellan gum and additionally supplemented with 500 mg/L streptomycin, 100 mg/L ampicillin and 20 mg/L cycloheximide. The cell suspension obtained after sonication was streaked on an M1H NAG ASW plate, incubated at 20 °C for six weeks and regularly checked for the presence of colonies. Colonies obtained were then subjected to 16S rRNA gene amplification and sequencing according to a previously published protocol (Rast et al. 2017). This step was included to check whether strains are members of the phylum *Planctomycetes*. Colonies of strains confirmed as members of the phylum *Planctomycetes* were re-streaked on M1H NAG ASW plates, which then served as a source for the inoculation of liquid cultures in M1H NAG ASW medium. After several days of cultivation, exponentially growing cells were used for subsequent cultivation experiments. Determination of the pH optimum for growth was performed by cultivation of strain Pla52n^T^ in M1H NAG ASW at 28 °C with 100 mM of the following buffers: 2-(*N*-morpholino)ethanesulfonic acid (MES) for pH 5.0-6.5, HEPES for pH 7.0-8.0, 3-(4-(2-hydroxyethyl)piperazin-1-yl)propane-1-sulfonic acid (HEPPS) for pH 8.0 and *N*-cyclohexyl-2-aminoethanesulfonic acid (CHES) for pH 9.0-10.0. Cultivations for determination of the temperature optimum for growth were performed in M1H NAG ASW medium at pH 7.5. Growth of the strain was measured as optical density at 600 nm (OD_600_). Maximal growth rates µ_max_ were obtained by determination of the slope in the plot of the natural logarithmic function of average OD_600_ values from biological triplicates against the cultivation time. The slope from at least five data points in the exponential growth phase was used as growth rate µ_max_ (in h^−1^). The generation time t_d_ (in h) was calculated using the equation t_d_ = ln(2)/µ_max_.

### Microscopy

Microscopic analyses included phase contrast light microscopy and field emission scanning electron microscopy (SEM) and were performed as previously described (Boersma et al. 2019).

### Genome information and antiSMASH analysis

Sequencing of the genome of strain Pla52n^T^ was conducted as part of a previous study (Wiegand et al. 2020). Genome and 16S rRNA gene sequence of strain Pla52n^T^ are available from GenBank under accession numbers GCA_007860045 and MK554582, respectively. Analysis of secondary metabolite-associated gene clusters was performed using antiSMASH bacterial version 5.1.2 with relaxed strictness and the following extra features enabled: KnownClusterBlast, ActiveSiteFinder and SubClusterBlast (Blin et al. 2019).

### Phylogenetic analysis

Maximum likelihood phylogeny was computed for strain Pla52n^T^, the type strains of all described planctomycetal species (assessed in May 2020) including strains of the family *Pirellulaceae* published and described in the recent year (Kallscheuer et al. 2019a, c, 2020a, b, c; Kumar et al. 2020; Peeters et al. 2020a, b; Rensink et al. 2020; Sandargo et al. 2020). Phylogenetic trees based on 16S rRNA gene sequences and multi-locus sequence analysis (MLSA) were calculated as previously described (Boersma et al. 2019). 16S rRNA gene sequences from *Opitutus terrae* (acc. no. AJ229235), *Kiritimatiella glycovorans* (acc. no. NR_146840) and *Lentisphaera araneosa* (acc. no. NR_027571) were used as outgroup in the 16S rRNA gene sequence-based tree. Two members of the family *Planctomycetaceae*, *Planctopirus limnophila* and *Gimesia maris*, were used as outgroup in the MLSA-based tree.

Average nucleotide identities (ANI) were calculated using OrthoANI (Lee et al. 2016), average amino acid identities (AAI) using the aai.rb script of the enveomics collection (Rodriguez-R and Konstantinidis 2016) and percentage of conserved proteins (POCP) as previously described (Qin et al. 2014). The *rpoB* nucleotide sequences were taken from publicly available genome annotations and the sequence identities for a partial sequence fragment of 1200 bp expected to be amplified with the described primer set were determined according to Bondoso et al. (2013).

## Results and discussion

### Phylogenetic inference

**Fig. 1:**
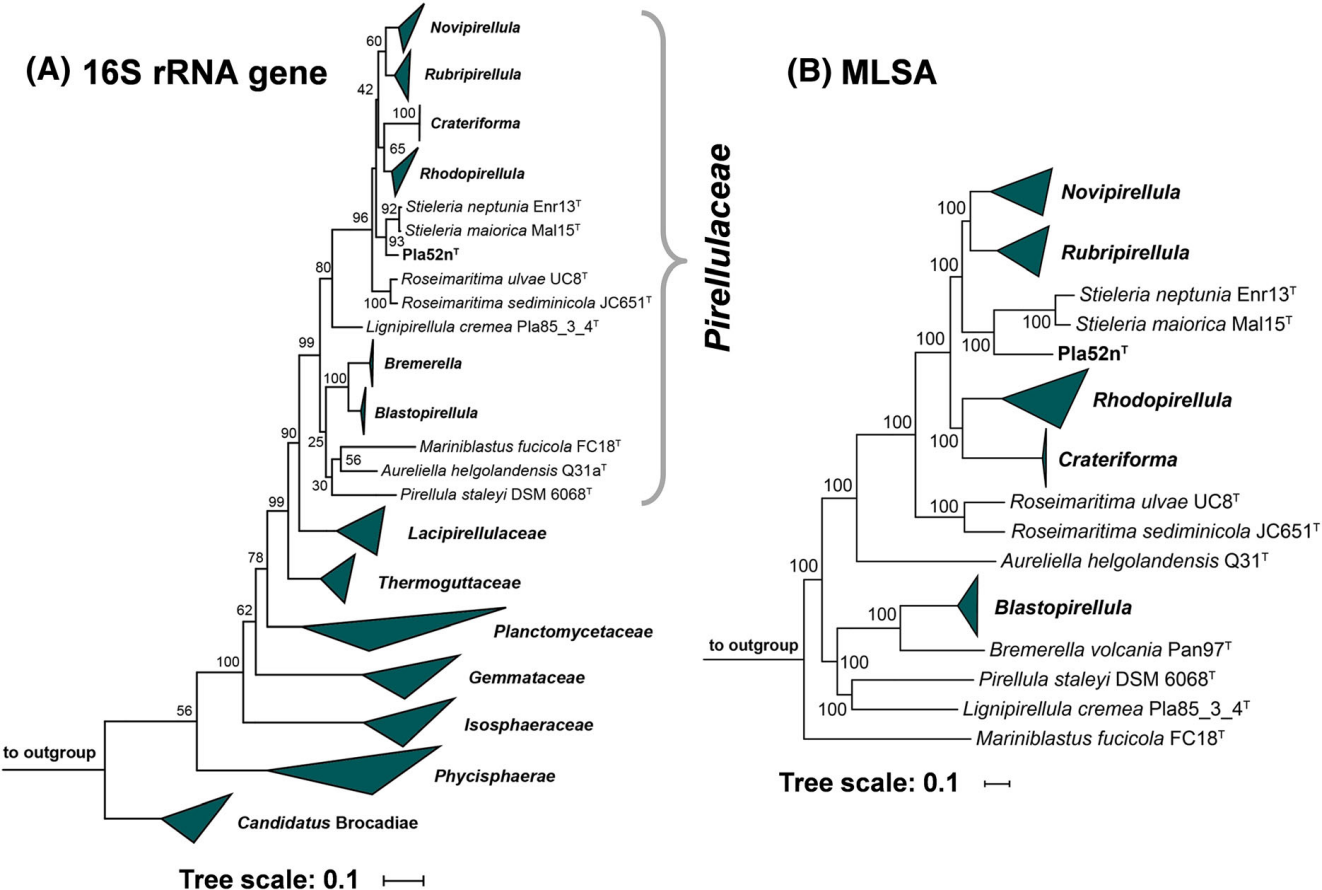
Maximum likelihood phylogenetic analysis of strain Pla52n^T^. Phylogenetic trees based on 16S rRNA gene sequences and MLSA were computed as described in the Materials and methods section. Bootstrap values after 1000 re-samplings (16S rRNA gene)/500 re-samplings (MLSA) are given at the nodes (in %). The outgroup in the 16S rRNA gene-based tree consists of 16S rRNA genes from three strains outside of the phylum *Planctomycetes* but part of the PVC superphylum. In the MLSA tree the genomes of *Planctopirus limnophila* and *Gimesia maris* (both family *Planctomycetaceae*) served as outgroup

**Fig. 2:**
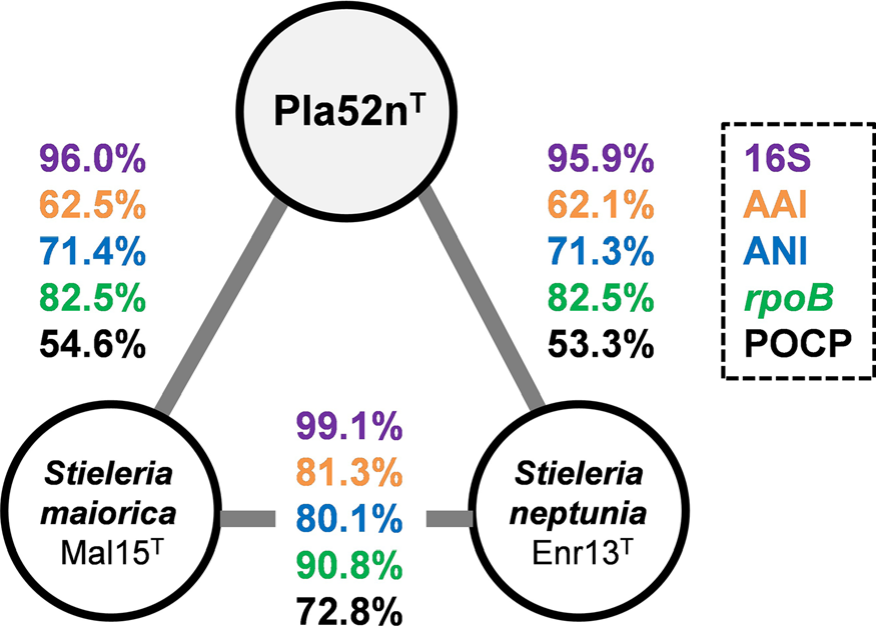
Analysis of phylogenetic markers used for the delineation of strain Pla52n^T^ from characterised species of the genus *Stieleria.* Analysed markers included 16S rRNA gene sequence similarity (16S), average amino acid identity (AAI), average nucleotide identity (ANI), identity of a 1200 bp fragment of the gene *rpoB* and percentage of conserved proteins (POCP)

Maximum likelihood phylogenetic trees based on 16S rRNA genes sequences and MLSA place strain Pla52n^T^ in the recently introduced family *Pirellulaceae* (Dedysh et al. 2019). In this family, strain Pla52n^T^ clusters monophyletically with two recently described species of the genus *Stieleria*, namely *S. maiorica* and *S. neptunia* (Kallscheuer et al. 2020a; Sandargo et al. 2020) (Fig. 1). Five investigated phylogenetic markers also identified these two species as the current closest relatives of strain Pla52n^T^ (Fig. 2). Comparison of the 16S rRNA gene sequence of strain Pla52n^T^ to those of *S. maiorica* Mal15^T^ and *S. neptunia* Enr13^T^ yielded similarities of 96.0% and 95.9%, respectively (Fig. 2). Both values are above the proposed genus threshold of 94.5% (Yarza et al. 2014) but below the species threshold of 98.7% (Stackebrandt and Ebers 2006), indicating that strain Pla52n^T^ is a member of the genus *Stieleria*, but does not belong to either of the two described species. This finding is in accordance with results obtained during an analysis of additional phylogenetic markers, including AAI, POCP and *rpoB* similarity when applying the proposed genus thresholds of 60%, 50% and 75.5–78%, respectively (Kallscheuer et al. 2019c; Konstantinidis and Tiedje 2005; Qin et al. 2014) (Fig. 2). In addition, none of the values obtained during comparison of strain Pla52n^T^ with its close neighbours were found to be above the species threshold for AAI and ANI of 95% (Kim et al. 2014; Konstantinidis and Tiedje 2005) and for *rpoB* of 96.3% (Bondoso et al. 2013). The conclusion that strain Pla52n^T^ belongs to a novel species within the genus *Stieleria* is thus supported by all analysed markers.

### Morphological and physiological analyses

**Fig. 3:**
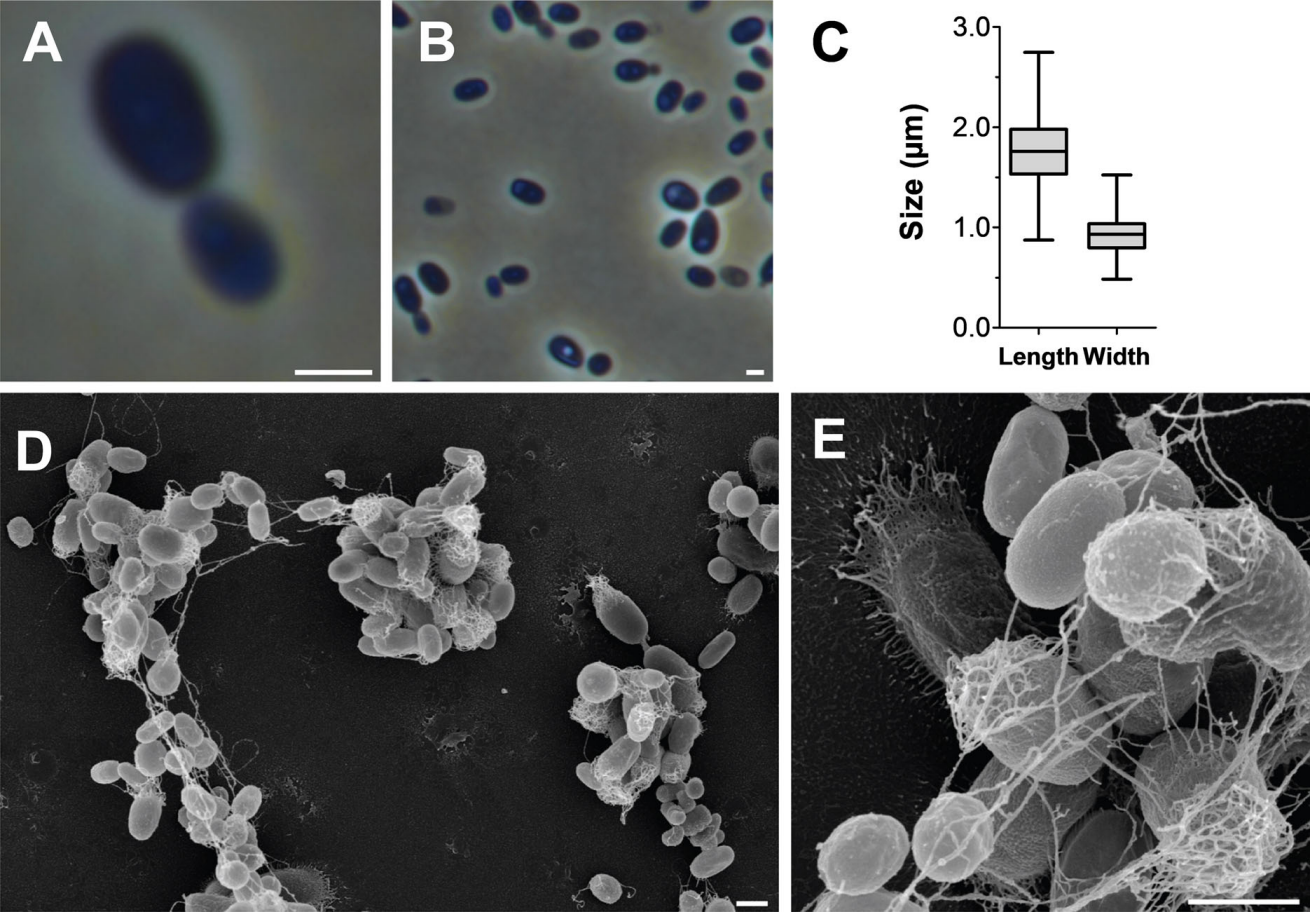
Microscopy images and cell size plot of strain Pla52n^T^. The mode of cell division (**a**) and a general overview of cell morphology (**b**, **d**, **e**) is shown in the micrographs, respectively. For determination of the cell size (**c**) at least 100 representative cells were counted manually or by using a semi-automated object count tool

**Fig. 4:**
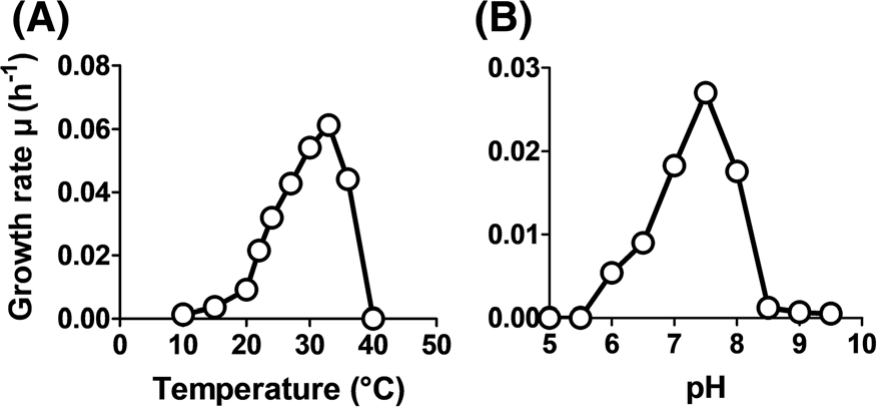
Temperature and pH optimum of strain Pla52n^T^. The graphs show the average growth rates obtained from cultivation in M1H NAG ASW medium in biological triplicates. Cultivations at different pH values were conducted at 28 °C and cultivations at different temperatures were performed at pH 7.5

Based on the phylogenetic position of strain Pla52n^T^, its phenotypic and genomic characteristics were compared to the two *Stieleria* species (Figs. 3, 4; Table 1). Cells of strain Pla52n^T^ have an average size of 1.8 ± 0.3 × 0.9 ± 0.2 µm (Fig. 3a, c), which is an elongated shape compared to more roundish or pear-shaped cells of *S. neptunia* Enr13^T^ and *S. maiorica* Mal15^T^. The shape of mature Pla52n^T^ cells turned out to vary from ovoid to round grain rice-shaped and is less uniform compared to the other two strains (Fig. 3b, d, e), which is reflected in the proposed name of the novel species represented by the type strain Pla52n^T^. All three compared strains either occur as single cells or form clusters, however, while *S. maiorica* and *S. neptunia* form larger aggregates, strain Pla52n^T^ tends to form rosettes which often assemble to short chains (Fig. 3d). Planktonic cells of all three strains are motile and at least for *S. maiorica* a clear lifestyle switch with sessile mother cells and swarming motile daughter cells was observed. Cells of the three strains contain crateriform structures and lack a stalk or holdfast structure. In case of strain Pla52n^T^, matrix or fimbriae originates from one of the poles and forms a characteristic fibre cap. Colonies of the strain have a light orange pigmentation, a quite rare pigmentation amongst characterised strains in the phylum, while the other two species have the more common pink pigmentation. Strain Pla52n^T^ can thus be clearly distinguished from the two described species, even with the naked eye. All three strains are aerobic heterotrophs and can grow up to temperatures of 35–37 °C. The temperature optimum of strain Pla52n^T^ (33 °C) (Fig. 4) falls between the optima of *S. maiorica* Mal15^T^ (35 °C) and *S. neptunia* Enr13^T^ (28 °C). Optimal growth is observed at pH 7.5 for all three strains. During laboratory-scale shaking flask cultivations in M1H NAG ASW medium, strain Pla52n^T^ reached a maximal growth rate of 0.061 h^−1^ (generation time of 11 hours, Fig. 4). Its growth rate is slightly higher than that observed for *S. neptunia* Enr13^T^ (0.054 h^−1^, t_d_ = 13 h), but considerably lower compared to *S. maiorica* Mal15^T^ (0.093 h^−1^, t_d_ = 7.5 h).

**Table 1:** Comparison of phenotypic and genomic features of strain Pla52n^T^ with its current closest relatives of the genus *Stieleria*

Characteristic	Pla52n^T^	*Stieleria neptunia* Enr13^T^	*Stieleria maiorica* Mal15^T^
*Phenotypic features*			
Length (µm)	1.8 ± 0.3	1.6 ± 0.1	1.9 ± 0.2
Width (µm)	0.9 ± 0.2	1.1 ± 0.1	1.4 ± 0.2
Shape	Ovoid to round grain rice-shaped	Round grain rice-shaped	Round to pear-shaped
Aggregates	Yes	Yes	Yes
Colony colour	Light orange	Pink	Pink
Division	Polar budding	Polar budding	Polar budding
Dimorphic life cycle	n.o.	n.o.	Yes
Temperature range (optimum) (°C)	15–36 (33)	9–35 (28)	11–37 (35)
pH range (optimum)	6.0–8.0 (7.5)	6.5–9.0 (7.5)	5.5–9.0 (7.5)
Relation to oxygen	Aerobic	Aerobic	Aerobic
Motility	Yes	Yes	Yes
Crateriform structures	Yes, at one pole	Yes, at one pole	Yes, at one pole
Fimbriae	Matrix at budding pole	Matrix or fimbriae	Matrix or fimbriae
Capsule	n.o.	Yes	n.o.
Stalk	n.o.	n.o.	n.o.
Holdfast structure	n.o.	n.o.	n.o.
*Genomic features*			
Genome size (bp)	9,586,696	10,975,817	9,894,293
Plasmids	n.o.	No	No
G + C content (%)	56.0	58.9	59.3
Completeness (%)	98.28	98.28	98.28
Contamination (%)	3.45	1.72	2.59
Coding density (%)	87.22	85.98	86.95
Total genes	7094	7904	7016
Genes/Mb	740	720	710
Giant genes	12	14	13
Protein-coding genes	6998	7797	6920
Protein-coding genes/Mb	730	710	699
Hypothetical proteins	3223	3425	2897
tRNA genes	80	99	81
16S rRNA genes	1	3	3

The genome analysis is based on GenBank accession numbers GCA_007860045 (strain Pla52n^T^), CP037423 (*Stieleria neptunia* Enr13^T^) and CP036264 (*Stieleria maiorica* Mal15^T^). Abbreviations: n.o. not observed

### Genomic characteristics of strain Pla52n^T^

The genome of strain Pla52n^T^ has a size of 9.59 Mb and a DNA G + C content of 56%. With such values the strain has currently the smallest genome and lowest G + C content in the genus *Stieleria* (Table 1). Its genome harbours 7094 genes, of which 6998 are putatively protein-coding. Automated gene annotation yielded 3223 genes coding for hypothetical proteins or proteins of unknown function, accounting for 46% of the total number of annotated proteins in the genome. These values fall within the range of 40–55% hypothetical proteins found to be encoded in most planctomycetal genomes sequenced so far and is comparable to the other two *Stieleria* species (42–43%). Given the relatively large genomes of the three strains, the presence of giant genes (with open reading frames > 15 kb) is not surprising. Strain Pla52n^T^ harbours 12 such genes, a number comparable to the two strains used for comparison (13–14 giant genes). An in silico analysis of the encoded proteins (> 5000 aa) based on domains detected by InterPro scan points towards a role as adhesion proteins or extracellular proteins with glycosyl hydrolase activity (Table 2). Plasmids were not observed in the genomes within the genus *Stieleria*. Strain Pla52n^T^ harbours a single copy of the 16S rRNA gene, whereas 3 copies can be found in the other two strains.

**Table 2:** Large proteins encoded by giant genes (> 15 kb) in strain Pla52n^T^

Locus tag	Protein annotation	Length (aa)	Putative function based on detected protein domains
Pla52n_07180	Cadherin domain protein	6975	Carbohydrate-binding, interaction of cells with extracellular matrix, cadherin-like adhesion protein, calcium-binding
Pla52n_11690	Cadherin-like domain-containing protein	10,563	Cadherin-related adhesion protein, glycosyl hydrolase activity, calcium-binding
Pla52n_17480	Tandem-95 repeat protein	6271	Glycosyl hydrolase activity, calcium-binding, cadherin-like adhesion protein
Pla52n_17490	Uncharacterized protein	7630	Polysaccharide hydrolysis, related to pectin lyase
Pla52n_17500	Matrixin	6858	Glycosyl hydrolase activity, calcium-binding, cadherin-like adhesion protein
Pla52n_23610	Tandem-95 repeat protein	13,990	Cadherin-related adhesion protein, cell surface protein, exopolysaccharide recognition, calcium-binding
Pla52n_27600	Putative Ig domain protein	5448	Cadherin-like adhesion protein, calcium-binding
Pla52n_42320	Uncharacterized protein	5541	Glycosyl hydrolase activity
Pla52n_57880	Tandem-95 repeat protein	7410	Glycosyl hydrolase activity, calcium-binding
Pla52n_57950	PKD domain-containing protein	8930	Glycosyl hydrolase activity, carbohydrate-binding, Integrin-related adhesion
Pla52n_59480	Hypothetical protein	9244	Extracellular protein, calcium-binding
Pla52n_68570	Tandem-95 repeat protein	6872	Cadherin-like adhesion protein, carbohydrate-binding, extracellular signalling, toxin system

The putative function of the gene is based on the analysis of protein domains detected by InterPro scan. The proteins in the table are sorted by the gene locus tag

### Analysis of gene clusters putatively involved in the production of secondary metabolites

Given the relatively large genomes of 9.6–11.0 Mb observed for the hitherto characterised species in the genus *Stieleria*, they are probably amongst the talented producers of secondary metabolites in the phylum *Planctomycetes* as confirmed by recently published studies (Kallscheuer et al. 2020a; Sandargo et al. 2020). We thus analysed the three genomes using antiSMASH to check for gene clusters potentially related to secondary metabolite production (Table 3). The analysis indicated that the three strains Pla52n^T^, *S. neptunia* Enr13^T^ and *S. maiorica* Mal15^T^ harbour a total number of 9-11 of such clusters. These numbers are in the upper range when considering the range of 1-13 clusters identified by antiSMASH in strains belonging to the phylum *Planctomycetes* (Wiegand et al. 2020). *S. neptunia* Enr13^T^ and *S. maiorica* Mal15^T^ harbour *N*-acyl amino acid synthase-encoding genes, which are most likely involved in the biosynthesis of stieleriacines, a class of *N*-acylated tyrosine derivatives found to be produced by these two strains (Kallscheuer et al. 2020a; Sandargo et al. 2020) (Table 3). However, in the still non-closed genome of strain Pla52n^T^ we could not identify genes coding for putative *N*-acyl amino acid synthases, which might indicate that strain Pla52n^T^ does not produce stieleriacines. This in not entirely unlikely given that strain Pla52n^T^ is more distantly related than the type strains of the other two *Stieleria* species (Fig. 2). Stieleriacine production is thus not necessarily a conserved feature within the genus *Stieleria*.

**Table 3:** Analysis of gene clusters putatively involved in secondary metabolite biosynthesis in strain Pla52n^T^ and its close relatives

Compound class	Pla52n^T^	*Stieleria* *neptunia* Enr13^T^	*Stieleria* *maiorica* Mal15^T^
Terpene	2	2	2
Type I PKS	3	1	1
Type II PKS	0	0	0
Type III PKS	1	1	1
NRPS	2	2	3
Type I PKS-NRPS	2	1	1
Bacteriocin	0	1	0
*N*-acyl amino acid	0	3	1
Ectoine	1	0	0
Total	11	11	9
Genome size (Mb)	9.59	10.98	9.89

The genome analysis was performed using antiSMASH version 5.1.2 and is based on GenBank accession numbers GCA_007860045 (strain Pla52n^T^), CP037423 (*Stieleria neptunia* Enr13^T^) and CP036264 (*Stieleria maiorica* Mal15^T^)

*PKS* polyketide synthase, *NRPS* non-ribosomal peptide synthetase

Other putative clusters associated with secondary metabolite production are related to polyketide and non-ribosomal peptide biosynthesis (Table 3). Two genes or clusters relevant to terpenoid production are likely involved in the production of carotenoids, as indicated by the orange or pink pigmentation of the *Stieleria* species.

Collectively, the polyphasic analysis justifies delineation of strain Pla52n^T^ from characterised species in the genus *Stieleria*. Thus, we propose to assign the novel isolate to a novel species, for which we propose the name *Stieleria varia* sp. nov.

#### *Stieleria varia* sp. nov

*Stieleria varia* (va’ri.a. L. fem. adj. *varia* varied; corresponding to the varying size of the cells).

Cells are ovoid to round grain rice-shaped (average size: 1.8 ± 0.3 µm × 0.9 ± 0.2 µm; cell size and shape are not uniform), occur as single cells or rosettes, which tend to form short chains. Crateriform structures are formed on one cell pole, while cells lack a stalk or holdfast structure. Matrix or fimbriae are formed at the budding pole. The species is heterotrophic, aerobic, mesophilic and neutrophilic. Daughter cells are motile. Optimal growth is observed at 33 °C and pH 7.5. Colonies have a light orange pigmentation. The type strain genome has a DNA G + C content of 56.0%.

The type strain is Pla52n^T^ (= LMG 29463^T^ = VKM B-3447^T^, synonym: Pla52neu), isolated from wood particles in the Baltic Sea in September 2014. The type strain has a genome size of 9,586,696 bp (GenBank Accession Number GCA_007860045).
